# Effects of Some Common Food Constituents on Cardiovascular Disease

**DOI:** 10.5402/2011/397136

**Published:** 2011-06-16

**Authors:** Yaling Yang, Sze Wa Chan, Miao Hu, Richard Walden, Brian Tomlinson

**Affiliations:** ^1^Department of Medicine and Therapeutics, The Chinese University of Hong Kong, Hong Kong; ^2^Centre for Clinical Pharmacology, University College London, London WC1E 6JF, UK

## Abstract

Cardiovascular diseases are the major cause of morbidity and mortality worldwide, and there is considerable interest in the role of dietary constituents and supplements in the prevention and treatment of these disorders. We reviewed the major publications related to potential effects on cardiovascular risk factors and outcomes of some common dietary constituents: carotenoids, flavonoid-rich cocoa, tea, red wine and grapes, coffee, omega-3 fatty acids, and garlic. Increased intake of some of these has been associated with reduced all-cause mortality or reduced incidence of myocardial infraction, stroke, and hypertension. However, although the evidence from observational studies is supportive of beneficial effects for most of these foodstuffs taken as part of the diet, potential benefits from the use of supplements derived from these natural products remain largely inconclusive.

## 1. Introduction

Cardiovascular disease (CVD) has been the major cause of morbidity and mortality in developed countries over the last several decades, and the prevalence is increasing dramatically in China and India and other developing countries. The underlying atheromatous vascular disease manifests as coronary heart disease (CHD), cerebrovascular disease, peripheral vascular disease, and subsequent development of heart failure and cardiac arrhythmias. The major risk factors for these disorders have been recognised for many years and include high levels of low-density lipoprotein (LDL) cholesterol, smoking, hypertension, diabetes, abdominal obesity, psychosocial factors, insufficient consumption of fruits and vegetables, excess alcohol, and lack of regular physical activity, and it was reconfirmed recently in the INTERHEART study that these conventional risk factors accounted for over 90% of the population attributable risk for myocardial infarction (MI) [1]. 

The management of the major risk factors with conventional drugs is effective in reducing cardiovascular events, and this is supported by extensive evidence from clinical trials. Recently, there is growing awareness of the place of dietary factors and herbal medicines in the prevention of CVD and the possibility of their use in treatment. Much of this interest centres on the antioxidant vitamins and the antioxidant properties of food constituents and herbal materials, but some herbal materials may also improve conventional cardiovascular risk factors or have antithrombotic effects. The 2005 US Dietary Guidelines and the American Heart Association have recommended the Diet Approaches to Stop Hypertension (DASH) diet, but most Americans are not following these guidelines and their daily consumption of fruit and vegetables are far short of the recommended targets [1, 2]. In 2009, it was estimated that only 32.5% of adults in the United States consumed fruit two or more times per day and 26.3% consumed vegetables three or more times per day [3]. In this paper we shall focus on some of the common food constituents which have been thought to have beneficial effects on CVD and will discuss some of the potential mechanisms and results from large clinical trials and meta-analyses.

## 2. Common Potentially Beneficial Food Constituents

Electronic literature searches were performed using MEDLINE, ISI Web of Knowledge, SCOPUS, and Science Direct (published from 1970 to 2010). The search terms used were cardiovascular diseases, clinical trial, meta-analysis, systematic review, carotenoids, cocoa, tea, red wine, grapes, coffee, omega-3 fatty acids, and garlic. A total of 2270 articles were identified. The bibliographies of all articles thus located were also scanned for further relevant references. Three authors (Y. Yang, S. W. Chan, and B. Tomlinson) extracted all articles independently and evaluated the quality of the studies and strength of the evidence of clinical impact using the same standards. Based on the relevance, strength, and quality of the design and methods, only the shortlist of the latest studies or representative findings were discussed below. Where meta-analyses were available, these have been discussed rather than the individual studies included in those analyses. Apart from some important mechanistic studies, *in vitro* and animal studies were generally excluded, as were articles written in languages other than English. 

### 2.1. Carotenoids

Numerous natural carotenoids are present in fresh fruits and vegetables, and some have been studied extensively in the prevention of CHD. Most carotenoids have free radical scavenging effect [4] and a potential to protect LDL cholesterol against oxidation [5, 6]. Carrots are a primary source of *β*-carotene. Elevated levels of serum *β*-carotene were associated with a lower risk of cancer and reduced overall mortality rates [7], and early observational studies reported an association between a high dietary intake of *β*-carotene and reduced incidence of CVD [8, 9]. In a case-control study, the risk of nonfatal acute MI in women was inversely associated with daily intake of *β*-carotene-containing diet [10]. In the Rotterdam study, a population-based cohort study in the elderly, dietary intake of *β*-carotene was inversely associated with the risk of MI [11]. Interestingly, in the American Health Professional's Study conducted in 39,910 US males, carotene intake was associated with a lower risk of CHD among current smokers but not nonsmokers [12].

Although many epidemiologic studies have reported an association between *β*-carotene and the risk of CVD, several large randomized trials did not reveal any reduction in CVD with *β*-carotene consumption. For instance, the MRC/BHF Heart Protection Study showed no benefit from *β*-carotene 20 mg daily, in combination with vitamin E 600 mg and vitamin C 250 mg, on morbidity or mortality in high-risk individuals [13]. In the *α*-tocopherol and *β*-carotene (ATBC) study conducted in 1,862 male smokers who had a previous MI, there were no significant differences in the number of major coronary events between any supplementation group and the placebo group. Moreover, the risk of fatal CHD was increased in the *β*-carotene and combined *α*-tocopherol and *β*-carotene groups compared to the placebo group [14]. Likewise, the Women's Antioxidant Cardiovascular Study (WACS) found no CVD risk reduction in women at high risk with *β*-carotene 50 mg every other day, or with vitamin C 500 mg daily or vitamin E 600 IU every other day [15]. The prospective evaluation of the relation between vegetable intake and CHD risk in the Physicians' Health Study concluded that the consumption of vegetables rich in carotenoids was associated with a reduced risk of CHD [16], but after 12 years of followup there was no impact from supplementation of *β*-carotene 50 mg alternate days on CVD, cancer, or overall mortality among primarily nonsmokers [17].

Lycopene, one of the most common carotenoids in the human diet, has twice the antioxidant activity of *β*-carotene [18]. Tomatoes are the best source of lycopene, which is the focus of research as a precursor to vitamin A. A diet rich in tomatoes, tomato products, and lycopene is associated with a lower risk of CHD [19]. Epidemiological studies and supplementation clinical trials suggested a reduction in CVD risk but not all studies have confirmed this. A multicentre case-control study suggested that lycopene may contribute to the protective effect of vegetable consumption on MI risk [20]. The Kuopio Ischaemic Heart Disease Risk Factor Study conducted in 1,028 middle-aged men in eastern Finland showed that subjects with low concentration of serum lycopene concentration had a significantly higher mean intima-media thickness of the common carotid artery (CCA-IMT) and higher maximal CCA-IMT than did the other men [21]. Conversely, in the Physicians' Health Study, no association between increasing concentrations of plasma lycopene and the risk of CVD was found [22]. A recent review of the controlled clinical studies with lycopene in well-defined subject populations found no definite evidence for CVD prevention [23]. Representative observational and intervention studies on the association of carotenoids with the risk of CVD are summarized in Table 1.

Apart from carotenoids and lycopene, many fruits and vegetables are also rich in glutathione. Glutathione and glutathione-1 peroxidase provide important antioxidant effects that may prevent CVD. A prospective study conducted in 636 patients with suspected coronary artery disease (CAD), with a median followup period of 4.7 years (maximum, 5.4), suggested that increasing glutathione-1 peroxidase activity might lower the risk of cardiovascular events [24]. A thorough review on the relations between plasma glutathione levels and CVD suggested that reduced plasma total glutathione levels are a risk factor for CVD especially for cerebral small vessel disease [25].

Currently, it seems logical that a higher dietary intake of fruit and vegetables rich in carotenoids may play a role in the prevention of morbidity and mortality associated with CVD. However, the evidence that specific supplements are beneficial is controversial, and the underlying mechanisms are not clear. Further studies are in progress to determine the usefulness of the consumption of carotenoids in the prevention and treatment of CVD.

### 2.2. Flavanol-Rich Cocoa

Flavanol-rich foods are common in the spectrum of the human diet and flavanols are present in items such as wine, tea, various fruits, and certain vegetables. Among these, cocoa and cocoa-derived products, such as cocoa powder and chocolate, are representative foods containing natural flavanols that have received much attention. Cocoa and cocoa-derived products are derived from the fermented, roasted, and industrially processed seed of the *Theobroma cacao* tree. The Kuna Indians in Panama have a very high intake of flavanol-rich cocoa, which may be related to them having low blood pressure levels, reduced frequency of cardiovascular events, and longer life expectancy than other Panamanians [26]. In cocoa and cocoa products, the flavanols include monomeric forms (catechins) and polymer forms (procyanidins). Monomers bind together by links between C4 and C8 and form dimers, and oligomers even up to decamers [27]. Procyanidins, also known as condensed tannins, combine with salivary proteins which cause the bitterness of cocoa as well as the astringent character of some fruits [28]. The major cocoa catechins include (+)-catechin, (−)-epicatechin, (+)-gallocatechin, and (−)-epigallocatechin. These monomers bind together to form procyanidin B1, procyanidin C1, procyanidin D, and so forth. 

Data from cell culture and animals studies suggest that cocoa may have antioxidant and anti-inflammatory effects [37]. An in vitro study examined nitric oxide (NO) levels in human umbilical vein endothelial cells under different treatment conditions. It found that epicatechin, a flavan-3-ol, scavenged free radicals, inhibited NADPH oxidase activity, and preserved the bioavailability of NO [38]. An animal study carried out on rabbit aortic rings showed that polymeric procyanidins derived from cocoa produced an endothelium-dependent relaxation which was mediated by activation of endothelial NO synthase [39]. In another study using cultured endothelial cells, cocoa decreased the activity of arginase which augmented the local levels of L-arginine, the precursor for NO synthesis [40]. It has been demonstrated that oral administration of L-arginine improves endothelium-dependent dilation in the forearm conduit arteries in patient with hypercholesterolemia [41]. In a prospective, double-blind, randomized crossover trial, long-term oral administration of L-arginine improved endothelium-dependent dilation and reduced monocyte adhesion to the endothelium in young men with coronary disease [42]. 

Accumulating epidemiological evidence suggests that flavanol-rich food such as cocoa has potential cardioprotective effects which might be attributed to improvements of cardiovascular risk factors (Table 2). In healthy male adults, daily intake of flavanol-rich cocoa drink over 1 week produces a sustained increase in FMD [43]. This long-term result on FMD is probably induced by elevated level of endothelial NO synthase (eNOS), which is further corroborated by in vitro data. In a double-blind, randomized study, consumption of chocolate improved coronary vascular function and reduced platelet adhesion, and these beneficial effects were coupled with reduced serum oxidative stress measures and changes in serum epicatechin concentration [44]. 

A prospective cohort study from Sweden with followup of 31,823 women over 9 years concluded that moderate habitual chocolate intake was associated with a lower incidence of heart failure. However, the protective association was not observed with greater intake of chocolate per day [31]. An observational study in a cohort of 19,357 German adults with followup of 8 years revealed that chocolate consumption lowered the risk of the combined outcome of MI and stroke which was partially due to reduced blood pressure [29]. It has also been reported that chocolate consumption is correlated with lower cardiac mortality in patients who survived a previous acute MI [30]. 

A recent meta-analysis, which included ten controlled studies involving normal adults or patients with hypertension and treated with cocoa products for a short-term ranging from 2 to 18 weeks, concluded that flavanol-rich cocoa products taken for 2–18 weeks reduce both systolic (−4.5 mmHg) and diastolic (−2.5 mmHg) blood pressures [45]. Furthermore, a meta-analysis of randomized controlled trials showed that chocolate or cocoa produced both acute and chronic effects on FMD but not on LDL cholesterol and high-density lipoprotein (HDL) cholesterol concentrations [46]. Several trials indicate that cocoa or chocolate inhibits platelet function [47, 48].

Evidence for cardiovascular benefits of cocoa flavanols has come largely from short-term and uncontrolled studies and, therefore, additional well-designed, long-term clinical trials of cocoa supplementation are required [49]. The mechanisms described above may partially explain the positive effects of flavanol-rich foods on CVD. Whether cocoa may possess antioxidant activity requires further investigation. It is important to consider that many chocolate-containing products also contain large amounts of fat and sugar which might negate any potential benefit from the flavanol content of the product.

### 2.3. Tea

Tea is one of the most popular beverages worldwide. It contains polyphenols in amounts similar to those found in red grapes. Tea is the product of the leaves and leaf buds of *Camellia sinensis*. Tea can be classified into 3 main categories according to the degree of fermentation: fully-fermented black tea, semifermented Oolong tea, and unfermented green tea. The polyphenolic compounds, mainly catechins, contained in green tea are associated with its cardiovascular protective effect. The major tea catechins include (−)epicatechin (EC), (−)-epigallocatechin (EGC), (−)-epicatechin-3-gallate (ECG), and (−)-epigallocatechin-3-gallate (EGCG). Catechins inhibit the expression of inducible NO synthase (iNOS) [50, 51] and reduce inflammation and ROS-generating enzymes [52, 53]. They also induce apoptosis of monocytes [54], lower lipids levels [55], reduce oxidative stress [50], inhibit platelet aggregation [56], and decrease apolipoprotein (Apo) B levels and increase the ratio of ApoA-1/ApoB [57]. The galloyl group may also exert cardiovascular protective effects via multiple cellular pathways and transcriptional factors involved in the cardiovascular system [58]. 

The association of green tea consumption with cardiovascular protection has been well documented in observational studies (see Table 2). A long-term study performed in Japan showed that daily consumption of 10 cups green tea was associated with a reduction in cardiovascular mortality in men [33]. In the Ohsaki study, a population-based prospective cohort study, green tea consumption was associated with a reduction in CVD mortality [32]. A prospective cohort study in 6,358 Japanese followed up for 5 years revealed an inverse correlation of green tea consumption and the risk of stroke incidence [34]. This association was further confirmed by a meta-analysis on 9 studies involving 4,378 individuals from different countries [59]. However, in a study of 2,855 Japanese, no association between green tea consumption and cardiovascular mortality was observed [60]. In a review of randomized controlled trials on the association between green tea and CVD risk profiles, only 17 out of 30 studies have reported beneficial effects of green tea, 11 studies showed neutral effects whereas 2 studies concluded harmful effects [61]. 

The increase in blood pressure shortly (30–90 minutes) after green tea consumption has been examined in several studies [62–64]. It is noteworthy that the increase in blood pressure is even greater than that produced by the same amount of caffeine administered alone [64]. Conversely, long-term consumption of tea may have a beneficial effect on blood pressure. In a cross-sectional study of 218 Australian women over 70 years old, long-term regular consumption of tea was associated with a lower systolic blood pressure and lower diastolic blood pressure [65]. In a cohort study conducted in 1,507 Chinese over 20 years old in which the mean blood pressure was carefully multiadjusted, daily consumption of 123–599 mL or over 600 mL green or oolong tea reduced the risk of developing hypertension by 46% and 65%, respectively, compared with non-habitual tea drinkers [35]. However, a larger cross-sectional study conducted in 3,336 Japanese males showed that green tea consumption failed to alter blood pressure [66]. 

Consumption of green tea has also been associated with lower levels of total cholesterol and LDL cholesterol [46], but without effects on serum HDL cholesterol and triglycerides [67]. A double-blind, randomized, controlled study conducted in 40 overweight Japanese children suggested that 24-week consumption of a drink made from green tea effectively reduced the LDL cholesterol level although the effect may have been related to weight loss [68]. A randomized, double-blind, placebo-controlled study in 111 healthy adult volunteers revealed that consumption of green tea (standardized and defined decaffeinated) for 3 weeks reduced LDL cholesterol levels [69]. However, several studies failed to find a significant correlation between green tea consumption and HDL cholesterol levels [69–71]. 

The majority of studies with tea have used green tea, but a few studies have been done using black tea and oolong tea. Short- and long-term consumption of black tea was shown to reverse endothelial vasomotor dysfunction but did not reduce *ex vivo* platelet aggregation in patients with CHD [72, 73]. Potential protective effects of green tea and black tea against CVD and cancer were attributed to the polyphenolic compounds, particularly the catechin, epigallocatechin gallate (EGCG) in green tea, and theaflavins in black tea. The processing of tea to produce black tea results in the conversion of catechins into theaflavins and thearubigins, but these also appear highly potent in NO production and vasorelaxation [74]. The concentrations of catechins including EGCG in black tea are much lower than that in green tea [75] but the theaflavins in black tea also provide an antioxidant effect which in some studies is similar to that of green tea [76]. In an epidemiological study on 17,228 subjects (mean age, 59.5 years) initially free of CVD and cancer from the College Alumni Health Study, black tea consumption was not associated with a reduced risk of CVD [77]. In a prospective study of 76,979 healthy individuals aged 40–79 years in Japan, consumption of coffee, green tea, and oolong tea and total caffeine intake were all associated with reduced risks of mortality from CVD [78]. A small study performed in The Netherlands, which studied the use of dietary bioflavonoids, phenolic acids, and quercetin, showed that there was a reduction in the incidence of heart attack and sudden death in the elderly men aged 65–84 years with a higher flavonoid intake and one of the major sources of flavonoid intake (61%) was from tea [79].

### 2.4. Red Wine and Grapes

The traditional French diet is high in saturated fats, but residents of France have a lower incidence of CAD than Americans, the so-called French paradox [80]. The typical French diet includes regular intake of fresh fruit and vegetables that contain phytonutrients which have antioxidant effects and may retard atherogenesis and thrombosis. However, consumption of red wine may be another protective factor. The rich polyphenolic content of red wine has made it a popular subject for consideration in the possible prevention of cancer and CVD [81]. Daily consumption of mild to moderate (1-2 drinks) amounts of red wine is thought to produce beneficial effects on CVD, which have been considered by some to provide the basis for the French paradox. 

Red wine contains many polyphenolic compounds including nonflavonoids and flavonoids [82]. These compounds have antioxidant, anti-inflammatory, and potential antiatherogenic effects. A study of the antioxidant activity of red wine in volunteers showed that two glasses of red wine before food had antioxidant activity lasting for at least 4 hours [83]. Red wine increases antioxidant activity through the flavonoid-polyphenol effect. Grapes and wines also contain catechol compounds which possess antioxidative effects. 

Part of the benefit of red wine may be from the alcohol content. The INTERHEART study, a very large case-control study conducted in 15,152 patients who had experienced an acute MI and 14,820 age- and sex-matched controls, has shown that regular consumption of alcohol (3 or more times a week) had a cardioprotective effect against MI [1]. However, in the prospective Copenhagen Heart study, low to moderate consumption of wine was associated with lower mortality from CVD and cerebrovascular disease and other causes whereas beer consumption failed to affect mortality and a similar intake of spirits appeared to increase the risk [36] (Table 2). In a meta-analysis including 13 studies and 209,418 participants, moderate red wine intake reduced the atherosclerotic risk by 37% and there was a similar, but smaller, association with reduced risk in beer consumption studies, again suggesting that red wine may have an additional benefit beyond the alcohol content [84].

Moderate red wine consumption has beneficial effects on some cardiovascular risk factors, particularly HDL cholesterol and possibly fibrinogen, which are not seen with red grape extract so those benefits may be attributed to the alcohol content and it has been suggested that any beneficial effect of red wine compared to other alcoholic beverages may be related to other life-style confounders rather than the nonalcohol components of red wine [85]. Several small studies have shown beneficial effects from the other components of red wine. A double-blind, cross-over study conducted in 15 CAD patients showed that acute consumption of either 250 mL regular or alcohol-free red wine significantly improved the arterial stiffness and wave reflections [86]. Interestingly, administration of alcohol-free red wine produces an improvement in various CVD risk parameters in some other studies. In line with these results, two studies showed that acute consumption of alcohol-free red wine caused an improvement of brachial artery flow-mediated vasodilation [87, 88]. However, some studies failed to show any additional protective effects of red wine consumption compared with other types of alcoholic beverages [89, 90].

Several studies have examined the potential benefits of specific components in red wine such as resveratrol and oligomeric proanthocyanidins in reducing CVD risk. Resveratrol is mainly found in grape skin, and, thus, significant amounts of resveratrol are present in red wine. It has been suggested that it has cardioprotective effect since it activates platelet NO synthetase [91] and inhibits LDL oxidation [92–94], inflammation [95], production of reactive oxygen species [96], and platelet aggregation [97]. Evidence from animal studies suggests that resveratrol exerts it cardioprotective effect by attenuating the proinflammatory effects invoked by platelet-activating factor [98] and upregulating the expression for iNOS, eNOS, VEGF, and KDR [99]. The beneficial effects of moderate wine intake on ischaemic CVD may be explained by this [100]. Many studies on resveratrol have been conducted in cultured cells and animal models. However, there is currently no definite evidence that resveratrol has cardioprotective effects in humans, and as the oral bioavailability of resveratrol in man is extremely low it is difficult to assess the true physiological significance of resveratrol [101]. 

Oligomeric proanthocyanidins are free radical scavengers [102] which inhibit lipid peroxidation [103] and have anti-inflammatory and antiallergenic properties [104]. Like carotenoids, they are found predominantly in brightly coloured fruits and vegetables and represent a safe source of polyphenols and quercetin, the latter being believed to be particularly active in preventing LDL oxidation [105]. In a placebo-controlled cross-over study with quercetin 150 mg daily supplementation for 6 weeks in overweight or obese subjects with metabolic syndrome traits, there was a decrease in systolic blood pressure by 2.6 mmHg, a decrease in plasma concentrations of atherogenic oxidised LDL, and a small but significant decrease in serum HDL cholesterol concentrations, although the ratio of LDL : HDL-cholesterol was unchanged [106]. 

The Physician's Health Study did not show any association between intake of flavonoids and all CAD events [107]. The Kuopio Ischaemic Heart Disease Risk Factor Study concluded that a high intake of flavonols and the mean CCA-IMT were negatively associated [108]. The optimal amount and form of flavonoids in the diet are not known, and some of these compounds have rather low bioavailability. In spite of the uncertainties, many flavonoids are available as food supplements in doses as high as 500 and 1000 mg, an amount 10 to 20 times the daily intake of a typical vegetarian diet. With the currently available information, patients with CAD and those at risk of CVD may be encouraged to include moderate red wine intake in their diet, but current research findings do not support the use of supplemental flavonoids derived from grapes or red wine, and further prospective controlled studies are needed to identify whether such supplements may be beneficial.

### 2.5. Coffee

Coffee from the seeds of plants of the *Coffea* genus is the most important overall source of caffeine in adults although caffeine is also present in tea, chocolate, and certain soft drinks [109]. Coffee also contains other biologically active compounds, including chlorogenic acid, and the diterpene alcohols cafestol and kahweol, which may have long-term effects on risk factors for CHD. Acute intake of coffee has a number of unfavorable cardiovascular effects, including increases in blood pressure, circulating catecholamines, arterial stiffness, and impairment of endothelium-dependent vasodilation which are probably attributable to caffeine [110]. However, studies examining the association between coffee consumption and CHD have been inconclusive, which may reflect the complex mixture of compounds that may have either beneficial or harmful effects on the cardiovascular system [111]. 

Acute intake of coffee raises systolic and diastolic blood pressure and slightly reduces heart rate, which is probably due to the effect of caffeine antagonizing the adenosine A_1_ and A_2A_ receptors [112]. Coffee also has a cholesterol-raising effect which appears to be related to diterpenes present in boiled coffee, and this may contribute to the risk of CHD associated with unfiltered coffee consumption [113]. Coffee consumption is associated with higher plasma total homocysteine concentrations which may increase the risk of CVD, and it was shown that caffeine was only partly responsible for the homocysteine-raising effect of coffee [114]. However, several studies have shown that coffee intake, including decaffeinated coffee, was inversely associated with the risk of developing type 2 diabetes mellitus [115], and another study showed that potentially black tea, but not green tea, in addition to coffee may also reduce the risk of type 2 diabetes mellitus [116].

A study of the association between coffee consumption and risk of acute nonfatal MI found that the polymorphism in the cytochrome P450 1A2 (CYP1A2) enzyme resulting in the variant CYP1A2*1F with “slow” caffeine metabolism modified the association so that increased intake of coffee was only associated with an increased risk of nonfatal MI among individuals with slow caffeine metabolism, suggesting that caffeine was a major factor in the association [117]. Several studies have examined the relationship between coffee intake and cardiovascular events and mortality with conflicting results. A recent long-term followup of participants in the Health Professionals Followup Study and Nurses' Health Study found that regular coffee consumption was not associated with an increased mortality rate in either men or women and there appeared to be a modest benefit of coffee consumption on all-cause and CVD mortality [118]. A lower risk of CHD among moderate coffee drinkers might be due to antioxidants found in coffee.

### 2.6. Omega-3 Poly-Unsaturated Fatty Acids

Early reports of the very low incidence of CHD in Greenland Eskimos [124] which was believed to be related to the high intake of seafood containing long-chain omega (n)-3 polyunsaturated fatty acids (n-3 PUFA) prompted many studies in other populations that have supported the theory that marine n-3 PUFAs protect against thrombosis, atherosclerosis, and CHD. The 3 major dietary n-3 PUFAs include eicosapentaenoic acid (EPA), docosahexaenoic acid (DHA), and *α*-linolenic acid (ALA). Some prospective cohort studies and randomized control trials in secondary prevention have found that consuming fish or fish oil containing EPA and DHA was associated with a decreased cardiovascular death rate, whereas the consumption of vegetable oil-derived ALA may not be as effective [125], but not all studies results are consistent. One systematic analysis concluded that long-chain and shorter-chain omega 3 fats do not have a clear effect on total mortality, combined cardiovascular events, or cancer [126]. 

Various studies show that doses >3 g/d, EPA plus DHA can improve many CVD risk factors, including reducing plasma triglycerides, blood pressure, platelet aggregation, and inflammation, along with improving vascular reactivity. A recent review considered the possible difference between whole fish and fish oil supplements and concluded that there was an association between dietary intake of n-3 PUFAs or fish with a reduced risk of subclinical atherosclerosis [127]. A randomized controlled trial of patients awaiting carotid endarterectomy indicated that n-3 PUFAs from fish oil enhanced the stability of atherosclerotic plaques [128]. However, it has been suggested that the therapeutic effects on CVD mortality could be attributed to a suppression of fatal arrhythmias rather than stabilization of atherosclerotic plaques [125, 129]. 

A meta-analysis on the effect of n-3 PUFAs in fish oil on blood pressure, which examined 31 controlled trials, concluded that greater consumption of n-3 PUFAs was associated with greater reduction in blood pressure, particularly in hypertensive subjects and those with clinical atherosclerotic disease or hypercholesterolemia [130]. In a randomized open-label, blinded endpoint study, which evaluated 18,645 patients with a total cholesterol of 6.5 mmol/L or above, daily consumption of 1800 mg EPA reduced posttreatment LDL cholesterol, unstable angina, and nonfatal coronary events [123]. A systematic review of 47 studies concluded that daily consumption of EPA and/or DHA (average 3.25 g) produces a significant reduction of triglycerides levels but not total, HDL, or LDL cholesterol in hyperlipidemic subjects [131]. 

Accumulating evidence has suggested that consumption of n-3 PUFAs decreases the risk of cardiovascular mortality and sudden cardiac death especially in patients with a history of MI. In the diet and reinfarction trial (DART), which involved 2,033 men recovered from MI, there was a significant reduction of total mortality in subjects who consumed 2-3 portions of fatty fish daily [132]. The GISSI-Prevenzione trial, which examined 11,323 patients who had experienced MI within 3 months, showed that daily intake of 1 g n-3 PUFA supplementation was associated with a significant reduction in all-cause and cardiovascular mortality, especially risk of sudden cardiac death [119]. The subsequent analysis to assess the time course of the benefit of n-3 PUFAs in the GISSI-Prevenzione trial found the survival curves for n-3 PUFA treatment diverged early after randomization, and total mortality was significantly lowered after 3 months of treatment suggesting that this early benefit supported the hypothesis of an antiarrhythmic effect [120]. In the GISSI-HF trial, patients with chronic heart failure of New York Heart Association class II-IV receiving n-3 PUFA 1 g daily showed small but significant reductions in all-cause mortality and hospital admissions for cardiovascular reasons [122].

In a randomized, double-blind, placebo-controlled study which was conducted in patients treated with chronic haemodialysis, consumption of n-3 PUFAs did not reduce the risk of cardiovascular event and death [121]. However, some clinical trials with increased consumption of fish oil showed an increase in sudden death in men [133, 134]. A review on the pro- and antiarrhythmic properties of n-3 PUFAs suggested that n-3 PUFAs may be antiarrhythmic under conditions that favour triggered activity but may also facilitate reentrant arrhythmias leading to sudden death and advice to increase intake of n-3 PUFA supplements or fatty fish should be tailored to individual patients with respect to the arrhythmogenic mechanisms associated with the underlying pathology [135]. However, a recent systematic review and meta-analysis on the effects of fish oil DHA and EPA on mortality and arrhythmias, which examined 12 studies totalling 32,779 patients, concluded that fish oil supplementation was associated with a significant reduction in deaths from cardiac causes but had no effect on arrhythmias or all-cause mortality and the optimal formulations for EPA and DHA remain unclear [136].

Most recently, a large study from Denmark following 57,053 middle-aged men and women for 7.6 years found that a modest intake of fatty fish was associated with a lower risk of acute coronary syndrome (ACS) with benefits seen for intakes >6 g of fatty fish per day but no obvious additional benefit for higher intakes and no benefit from intake of lean fish [137]. There were few cases of ACS in women and no consistent associations with fish intake were observed in the women. Studies pertaining to the effects of n-3 PUFA supplements on cardiovascular risks are summarized in Table 3. Current recommendations from the American Heart Association are that everyone should eat oily fish twice per week for primary prevention and that people with established CHD should take 1 g/d of EPA and DHA from oily fish or supplements [138, 139].

### 2.7. Garlic (Allium sativum)

For centuries garlic has been valued for its medicinal properties. As an herbal medicine it has been more closely examined than many others. Much research has focused on garlic for preventing atherosclerosis. Multiple beneficial cardiovascular effects have been found including lowering of blood pressure, inhibition of platelet aggregation, enhancement of fibrinolytic activity, reduction in cholesterol and triglyceride, and protection of the elastic properties of the aorta [140]. 

The intact cells of garlic bulbs contain an odourless, sulphur-containing amino acid allinin. When garlic is crushed, allinin comes into contact with allinase which converts allinin to allicin. Fresh garlic releases allicin in the mouth during chewing. This has potent antibacterial properties is highly odoriferous and unstable. Ajoenes are the self-condensation products of allicin and appear to be responsible for garlic's antithrombotic action. It is generally considered that allicin and its derivatives are the active constituents of garlic's physiological activity. Dried garlic preparations lack allicin but contain both allinin and allinase. Since allinase is inactivated in the stomach, dried garlic preparations should have enteric coating so they pass unaltered through the stomach to the small intestine where allinin is enzymatically converted to allicin. Only few commercially available garlic preparations are standardised for their yield of allicin based on the allinin content [141]. 

The consumption of large quantities of fresh garlic (0.25 to 1.0 g/kg or about 5–20 average sized 4 g cloves) has been shown to produce certain beneficial effects [142]. In support of this, a recent double-blind, cross-over study in moderately hypercholesterolemic men comparing the effects of 7.2 g of aged garlic extract with placebo on blood lipid levels found a maximal reduction of 6.1% in total serum cholesterol levels and 4.6% in LDL cholesterol levels with garlic compared with placebo [143]. However, despite positive evidence from a number of trials, full endorsement of garlic for CVD is not forthcoming and many published studies have methodological shortcomings [142, 144–149]. Some of the problems were that trials were small, they lacked statistical power, they had inappropriate methods of randomization, they lacked dietary run-in periods, they were of short duration, or they failed to undertake intention-to-treat analysis. This has led to a cautious approach in the interpretation of previous meta-analyses [147]. One recent meta-analysis found that garlic reduces total cholesterol to a modest extent, an effect driven mostly by the modest reductions in triglycerides and there was no appreciable effect on LDL or HDL cholesterol [150].

Garlic has also been studied in hypertension, with no conclusive result [151]. A meta-analysis of 8 trials suggested some clinical value in patients with mild hypertension, but the evidence was insufficiently good to commend garlic for routine clinical therapy [149]. Garlic has been shown to have antiplatelet stickiness activity. This has been documented in vitro [152], and a new study examined the effect of consuming a clove of fresh garlic on platelet thromboxane production. After 26 weeks, serum thromboxane levels were reduced by about 80% [153]. Thus it may prove to be of benefit in the prevention of thrombosis. Another trial showed that long-term intake of 300 mg daily of standardised garlic powder for more than 2 years improved the elastic properties of the aorta [154]. In these ways garlic has shown several benefits to cardiovascular health and needs further study. Moderate garlic consumption causes few adverse effects other than bad odour. However, with more than 5 cloves daily, heartburn, flatulence, and other gastrointestinal disturbances have been reported. Allergic contact dermatitis has occurred, and patch testing is available when garlic allergy is suspected [155]. Due to its antithrombotic activity, garlic should be taken with caution in people on oral anticoagulants [156].

## 3. Research Needs

The clinical studies reviewed above are generally not supportive of the use of supplements of these food constituents although guidelines generally recommend increasing intake of fruits and vegetables which are rich in some of these materials. Omega-3 poly-unsaturated fatty acids may be one exception where supplements appear to be useful in people with established CHD. With the other dietary constituents which may have benefits in the prevention or treatment of CVD, the evidence from intervention trials is mostly not sufficient to support any definitive recommendations. Many of the trials have been too small, and different trials have often used different supplements with variable composition so the meta-analyses of such interventions may not always be considering the same active compounds. Many of these food substances do have ingredients with demonstrable pharmacological effects, but larger clinical trials with properly standardized materials are needed before any clear conclusions can be drawn. In addition, the potential interaction of some chemical components, especially flavonoids, with conventional drugs through mechanisms such as modulating ABC transporter expression may affect the absorption, distribution, and excretion of drugs. For instance, catechins, found in tea and red wine, may alternatively inhibit or enhance P-glycoprotein (ABCB1) function [157], and some of these compounds such as quercetin found in tea and red wine are substrates of P-glycoprotein [158]. These interactions could improve absorption of poorly absorbed drugs, but they could also lead to drug intoxication and interfere with drug excretion process so it is important to consider potential drug interactions if large doses of these materials are given as supplements.

## 4. Conclusions

Several common food constituents are thought to influence the development and progression of CVD, and this is supported by evidence of potentially beneficial biological actions and analyses from some observational studies. However, the evidence of beneficial effects in studies involving supplementation is generally inconclusive, apart from the use of omega-3 PUFA in patients with established CAD. Furthermore, excessive intake of some of the components which may be taken with these items, such as alcohol, saturated fat, and glucose, is likely to have harmful effects which may offset any benefit from the other active ingredients. Until more definitive results become available, the best approach is to recommend a well-balanced diet that includes adequate fruit and vegetables with moderate amounts of the food constituents discussed here. A summary of recommended lifestyle advice for primary and secondary prevention based on CVD risk groups is showed in Table 4. It is important to recognize that the current worldwide epidemic of obesity, diabetes and resulting CVD is related to excessive intake of calories and animal fats along with reduced physical activity and supplementation with any additional beneficial food material is unlikely to overcome these problems unless the underlying causes are addressed directly.

##  Conflict of Interests

The authors have no conflict of interests in relation to this paper.

## Figures and Tables

**Table 1:** Observational and intervention studies of the association of carotenoids with the risk of cardiovascular disease.

Authors	Type of study	Subjects	Methods	Findings
Cook et al. [15]	Randomized controlled factorial	8,171 female health professionals aged ≥40 with a history of CVD or ≥3 CVD risk factors with followup of 9.4 years	Vitamin C (500 mg/d), E (600 IU every other day), or *β*-carotene (50 mg every other day)	No association between vitamin C, E or *β*-carotene on cardiovascular events.
Heart Protection Study Collaborative Group [13]	Randomized placebo-controlled	20,536 UK adults aged 40–80 with CHD, OAD, or DM with followup of 5 years	*β*-carotene 20 mg/d, vitamin E 600 mg/d, and vitamin C 250 mg/d, or placebo	No association between *β*-carotene consumption and the 5-year mortality from, or incidence of, any type of vascular disease
Hennekens et al. [17]	Randomized, double-blind, placebo-controlled	22,071 US male physicians aged 40–84 without heart disease, stroke or cancer with followup of 12 years	*β*-carotene 50 mg/d or placebo	No association between *β*-carotene supplementation and the incidence of CVD, or death from all causes among primarily nonsmokers.
Klipstein-Grobusc et al. [11]	Community-based prospective	4,802 subjects aged 55–95	Questionnaire and interview with trained dietitian	An inverse association between high dietary *β*-carotene intake and risk of MI
Kohlmeier et al. [20]	Multicenter case-control	1,387 subjects from 10 European countries with MI or controls	Assay of 3 carotenoids in adipose tissue biopsies	Adipose tissue lycopene level was best predictor of MI risk
Rapola et al. [14]	Randomized, double-blind, placebo-controlled	1,862 male smokers aged 50–69 with previous MI with follow-up of 5.3 years	*α*-tocopherol (50 mg/d), *β*-carotene (20 mg/d), both, or placebo	Supplementation of *β*-carotene increased the risk of fatal coronary events
Rimm et al. [12]	Prospective	39,910 US male health professionals aged 40–75	Interview using questionnaire	Higher carotene intake was associated with lower risk of CHD only among current smokers
Rissanen et al. [21]	Cross-sectional	1,028 men aged 46–64 in eastern Finland	Blood assay and CCA-IMT measurement	Lowest quartile of serum lycopene had higher CCA-IMT
Sesso et al. [22]	Prospective nested case-control study	499 with CVD and 499 controls male US physicians	Blood assay and self-reported data to a standard questionnaire	No association between plasma lycopene and the risk of CVD
Tavani et al. [10]	Case-control study	433 Italian women with nonfatal AMI and 869 controls	Interview on dietary *β*-carotene intake using a structured questionnaire	The risk of AMI was inversely related to *β*-carotene intake for the highest quintile of intake compared to the lowest

AMI: acute myocardial infarction; CCA-IMT: intima-media thickness of the common carotid artery; CHD: coronary heart disease; CVD: cardiovascular disease; DM: diabetes mellitus; MI: myocardial infarction; OAD: occlusive arterial disease.

**Table 2:** Observational studies of the association of flavanol-rich food with the risk of cardiovascular disease.

Authors	Type of study	Subjects	Methods	Findings
*Cocoa*				
Buijsse et al. [29]	Prospective	19,357 Germans aged 35–65 years free of MI, stroke and not on antihypertensive medication with mean followup of 8 years	Interview using questionnaire on consumption frequency of 50 g milk/dark/white/unspecified chocolate bars	Highest quartile of chocolate consumption (mainly milk and dark chocolate) had lower relative risk of the combined outcome of MI and stroke
Janszky et al. [30]	Population-based case-control	1,169 nondiabetic and newly diagnosed first AMI patients with followup of 8 years	Questionnaire on consumption frequency of 50 g chocolate (ranging from never to twice or more per week)	Chocolate consumption reduced cardiac mortality in a dose-dependent manner
Mostofsky et al. [31]	Prospective	31,823 women aged 48–83 in Sweden without DM, HF or MI with followup of 9 years	Questionnaire on chocolate consumption frequency (ranging from never to ≥3 times/d)	Moderate (1–3 times/month) habitual chocolate intake was associated with a lower rate of HF hospitalization or death but there was no protection with intake ≥1 time/d

*Tea*				
Kuriyama et al. [32]	Population-based prospective	40,530 Japanese aged 40–79 without history of stroke, CHD, or cancer with followup of 11 years	Questionnaire on green tea consumption frequency (ranging from never to >5 cups/d)	Compared with those consumed <1 cup/d green tea, consumption of ≥5 cups/d had lower risk of all-cause and CVD mortality, and this effect was stronger in women
Nakachi et al. [33]	Prospective	8,497 residents in Japan aged over 40 with followup of 11 years	Questionnaire on green tea consumption frequency (ranging from <3 to over 10 cups/d)	Consumption of >10 cups/d green tea decreased the relative risk of death from CVD
Tanabe et al. [34]	Prospective	6,358 Japanese aged 40–89 without a history of stroke or heart disease with followup of 5 years	Questionnaire on tea consumption frequency (ranging from ≤ several cups a week to > 5 cups/d)	Green tea consumption of > several cups every 2-3 days was associated with reduced risk of total stroke incidence
Yang et al. [35]	Prospective	1,507 Taiwanese aged ≥20 without hypertensive history	Interview using questionnaire on green tea consumption frequency (ranging from nonhabitual <120 mL/d to ≥600 mL/d) and standard measurement of anthropometry and blood pressure	An inverse association between the newly diagnosed hypertension risk and habitual tea consumption

*Red wine and grapes*				
Gronbaek et al. [36]	Community-based prospective	13,285 subjects in Denmark aged 30–70 with followup of 10–12 years	Questionnaire on alcohol (beer, wine, or spirit) consumption	Low to moderate intake (3–5 glasses) of wine reduced the risk of death from CVD and cerebrovascular disease

AMI: acute myocardial infarction; CHD: coronary heart disease; CVD: cardiovascular disease; DM: diabetes mellitus; HF: heart failure; MI: myocardial infraction.

**Table 3:** Intervention studies of the association of omega-3 poly-unsaturated fatty acids consumption with the risk of cardiovascular disease.

Authors	Type of study	Subjects	Interventions	Findings
GISSI Prevenzione investigators [119, 120]	Multicenter, randomized, controlled, open-label, parallel	11,323 patients surviving recent (≤3 months) MI with followup of 3.5 years	Supplements of n-3 PUFA (1 g/d), vitamin E (300 mg/d), both, or none	Early protection of n-3 PUFA on all- cause mortality especially sudden cardiac death; over 3.5 years, supplementation with n-3 PUFA was associated with lower risk of death, nonfatal MI, and stroke.
Svensson et al. [121]	Randomized, double-blind, placebo-controlled	206 patients with CVD and treated with stable chronic hemodialysis with followup of 2 years	Supplements of n-3 PUFA (1.7 g/d), or control (olive oil)	Consumption of n-3 PUFA reduced the incidence of MI but had no effect on all- cause mortality or total cardiovascular event
Tavazzi [122]	Multicenter, randomized double-blind, placebo-controlled	6,975 patients aged ≥18 with chronic heart failure with followup for a median of 3.9 years	n-3 PUFA 1 g/d or placebo	Consumption of n-3 PUFA was associated with reduced all- cause mortality, hospital admissions for CVD
Yokoyama [123]	Randomized, controlled, open-label, blinded endpoint	18,645 patients with a total cholesterol of ≥6.5 mmol/L with followup of 5 years	Daily consumption of 1800 mg of EPA with statin or statin only	Daily consumption of EPA with statin reduced unstable angina and nonfatal coronary events.

CVD: cardiovascular disease; EPA: eicosapentaenoic acid; GISSI: Gruppo Italiano per lo Studio della Sopravvivenza nell'Infarto Miocardico; MI: myocardial infraction; n-3 PUFA: omega (n)-3 polyunsaturated fatty acids.

**Table 4:** Summary of recommended lifestyle advice based on cardiovascular risk groups.

CVD risk	Recommendations
High Risk : Clinically determined ≥20%*	Intensive lifestyle advice on a cardioprotective dietary pattern with a dietitian, physical activity, and smoking cessation interventions. Lifestyle advice should be given simultaneously with drug treatment.
Medium Risk : Calculated 10–20%	Specific individualized lifestyle advice on a cardioprotective dietary pattern, physical activity, and smoking cessation. This lifestyle advice should be given by the primary health care team for 3–6 months prior to initiating drug treatment.
Low Risk : Calculated <10%	General lifestyle advice on a cardioprotective dietary pattern, physical activity, and smoking cessation.

*People who have had a previous cardiovascular event (angina, MI, angioplasty, coronary artery bypass grafts, TIA, ischaemic stroke, or peripheral vascular disease) or people with certain genetic lipid disorders or people with DM and who are over 40 years.

Adapted from American Heart Association guidelines for primary prevention of cardiovascular disease and stroke: 2002 updates and New Zealand cardiovascular guidelines handbook [138, 159].
